# Chikungunya virus in dengue-suspected patients: Molecular evidence from the 2019 outbreak in Yangon, Myanmar

**DOI:** 10.1371/journal.pntd.0014258

**Published:** 2026-05-04

**Authors:** Merveille Kapandji, Htin Lin, Maurine Mumo Mutua, Qiang Xu, Ryosaku Oshiro, Catarina Harumi Oda Ibrahim, Micheal Teron Pillay, Kei Yamasato, Khine Mya Nwe, Muhareva Raekiansyah, Shyam Prakash Dumre, Kyaw Zin Thant, Wah Wah Aung, Aye Aye Khin, Hlaing Myat Thu, Takeshi Urano, Kouichi Morita, Yuki Takamatsu, Mya Myat Ngwe Tun

**Affiliations:** 1 Department of Virology, Institute of Tropical Medicine, Nagasaki University, Nagasaki, Japan; 2 Graduate School of Biomedical Sciences, Nagasaki University, Nagasaki, Japan; 3 Program for Nurturing Global Leaders in Tropical and Emerging Communicable Diseases, Graduate School of Biomedical Sciences, Nagasaki University, Nagasaki, Japan; 4 Department of Virology, National Institute of Biomedical Research, Kinshasa, Democratic Republic of the Congo; 5 Department of Medical Research, Ministry of Health, Yangon, Myanmar; 6 Department of Central Laboratory, GUIZHOU Provincial People’s Hospital, Guiyang, China; 7 DEJIMA Infectious Disease Research Alliance, Nagasaki University, Nagasaki, Japan; 8 Department of Vaccine Informatics, Institute of Tropical Medicine, Nagasaki University, Nagasaki, Japan; 9 Department of Tropical Viral Vaccine Development, Institute of Tropical Medicine, Nagasaki University, Nagasaki, Japan; 10 Central Department of Microbiology, Tribhuvan University, Kathmandu, Nepal; 11 Myanmar Academy of Medical Science, Yangon, Myanmar; 12 Center for Vaccines and Therapeutic Antibodies for Emerging Infectious Diseases, Shimane University, Izumo, Japan; University of Dhaka, BANGLADESH

## Abstract

**Background:**

Chikungunya virus (CHIKV) and dengue virus (DENV) frequently co-occur in Myanmar and present with overlapping symptoms, complicating diagnosis. During the 2019 dengue outbreak in Yangon, Myanmar, molecular data on CHIKV were limited among dengue-suspected patients and there were no publicly available CHIKV genome sequences from Yangon in international databases. To address this gap and potential diagnostic overlap, we investigated the prevalence of CHIKV infection and described the genomic characteristics of detected strains.

**Methods:**

Serum samples from 267 dengue-suspected patients collected in 2019 were screened for anti-CHIKV IgM and IgG by in-house ELISA and 211 samples with sufficient remaining volume were further analyzed by RT-qPCR, isolation of the virus, and whole-genome sequencing for mutation analysis.

**Results:**

CHIKV antibodies were found in 24.7% (66/267) of samples (IgM 3.4%, IgG 21.3%), and viral RNA was detected in 10.9% (23/211) of samples. Fifteen viral isolates were successfully obtained (7.1% of those tested), including two co-detections with DENV-2 by RT-PCR. All isolates belonged to the East/Central/South African genotype, Indian Ocean Lineage (ECSA-IOL), and clustered with strains from Thailand, China, and Mandalay, Myanmar. Whole-genome analysis identified 33 non-synonymous mutations across nonstructural and structural proteins, including mutations previously reported in regional ECSA-IOL strains such as E1:K211E and E2:V264A, with 11 amino acid changes not previously reported in available Myanmar reference sequences.

**Discussion:**

Serological and molecular findings indicate CHIKV circulation during the 2019 dengue outbreak in Yangon and highlight the limitations of single-target testing. Serological evidence indicate the presence of anti-CHIKV IgM and IgG antibodies, reflecting CHIKV exposure within the study population. Notably, all RNA-positive cases were seronegative for both IgM and IgG, a pattern consistent with the temporal dynamics of infection and the inherent constraints of serological detection in co-endemic settings. Molecular co-detection with DENV-2 and genomic findings highlight the potential value of multiplex diagnostic approaches in co-endemic settings.

**Conclusion:**

This study documents CHIKV detection and genomic characterization in dengue-suspected patients in Yangon and highlights the potential value of multiplex diagnostic approaches and continued genomic surveillance as broader public health considerations for arboviral detection in Myanmar.

## Author summary

Chikungunya virus (CHIKV) and dengue virus (DENV) are mosquito-borne viruses that often co-circulate in endemic regions and cause similar clinical symptoms, making accurate diagnosis difficult without laboratory confirmation. In Myanmar, while dengue is routinely monitored, surveillance for CHIKV is limited. During the 2019 dengue outbreak in Yangon, serum samples were collected from dengue-suspected patients. We re-examined these samples to investigate possible CHIKV co-circulation. Among 267 patients, 24.7% had antibodies against CHIKV, indicating evidence of exposure to the virus. CHIKV RNA was identified in 10.9% of 211 samples with sufficient volume for molecular testing, but only among those who were DENV NS1-negative. Infectious virus was successfully isolated in a subset of samples, including two cases of co-detection with DENV-2 by RT-PCR. These findings demonstrate CHIKV circulation during a dengue outbreak in Yangon and highlight the limitations of single-pathogen testing. Our results underscore the public health importance of integrated diagnostic approaches and continued genomic surveillance to improve detection and monitoring of arboviral infections in co-endemic regions.

## Introduction

Chikungunya virus (CHIKV) is an arthropod-borne virus (arbovirus) belonging to the genus *Alphavirus* within the family *Togaviridae* [1]. The virus is primarily transmitted through the bite of infected *Aedes aegypti* (Linnaeus) or *Ae. albopictus* (Skuse) mosquitoes [2,3]. CHIKV infection presents clinical features overlapping with other arboviral infections, including Dengue Virus (DENV) and Zika virus (ZIKV), complicating clinical diagnosis, particularly during concurrent outbreaks [4]. Laboratory tests are essential for distinguishing between DENV and CHIKV infections. The World Health Organization (WHO) recommends that all febrile patients should initially be tested for DENV, followed by CHIKV testing in DENV-negative cases [5]. Myanmar borders several countries with documented CHIKV circulation, including China, India, Bangladesh, Laos, and Thailand, where outbreaks have been reported [6,7]. Within Myanmar, CHIKV has been detected since 1976, with subsequent evidence of circulation and isolation of ECSA strains in Mandalay, including reports of DENV-CHIKV co-detection [5,8–10]. In contrast, evidence from Yangon remains limited to CHIKV viral RNA detection in healthy volunteers in 2019, with no corresponding characterization in dengue-suspected patients [11]. The absence of CHIKV isolation and genomic characterization from dengue-suspected patients in Yangon during the 2019 outbreak represents a key gap in understanding local CHIKV circulation and its genomic features. Given that CHIKV is a recognized global public health concern with substantial socio-economic impact [12,13], addressing this gap is important for improving epidemiological understanding in this setting. In Yangon, where CHIKV surveillance data remain limited and clinical presentations overlap with dengue, the extent of CHIKV circulation among dengue-suspected patients during the 2019 outbreak is not well defined. Therefore, the primary objective of this study was to assess CHIKV detection among dengue-suspected patients in Yangon during the 2019 outbreak using serological and molecular methods. Secondary objectives were to isolate viable CHIKV strains and perform whole-genome sequencing to characterize their phylogenetic relationships and genomic features.

## Methods

### Ethical statement

This study received clearance from the Ethics Review Committee on Medical Research Involving Human Subjects at the Department of Medical Research, Myanmar (Ethics/DMR/2017/068), and from the Institutional Review Board of the Institute of Tropical Medicine, Nagasaki University, Japan (170707205). Written informed consent was obtained from all participants and, where applicable, their legal guardians. Archived serum samples were collected under consent permitting future arboviral research and reuse. Retrospective chikungunya analyses were approved under the existing protocol by both ethics committees.

### Study population

**Fig 1:**
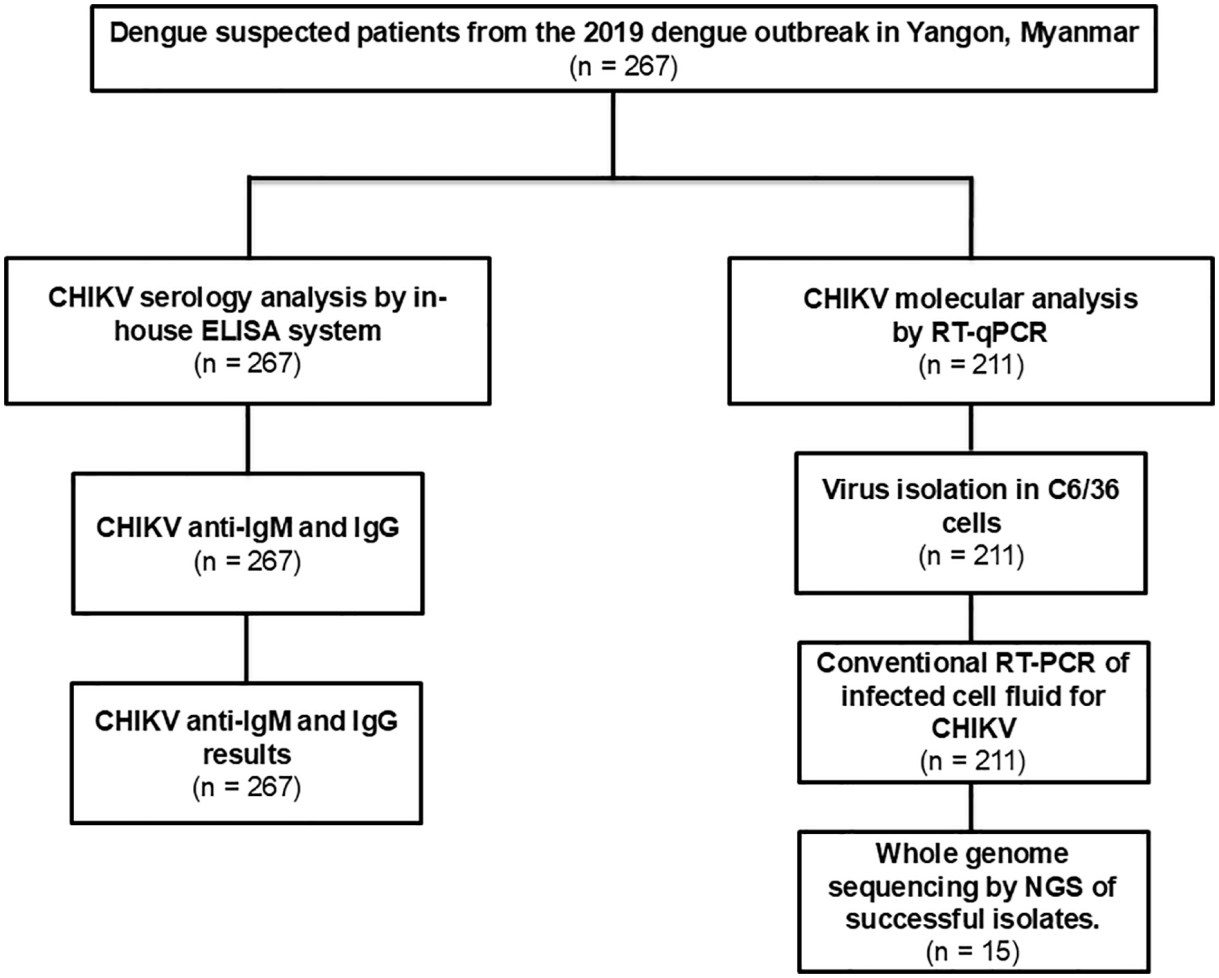
Flowchart illustrating the number of samples and laboratory analyses conducted in this study.

This retrospective study analyzed archived serum samples collected from dengue-suspected patients during the 2019 outbreak in Yangon, Myanmar, originally used in prior DENV studies [14,15]. A total of 267 patients were enrolled, samples were collected from children at Yangon Children’s Hospital and adults at Yangon General Hospital. The cohort was predominantly pediatric (≤15 years: 256/267; adults 16–45 years: 11/267), and findings therefore primarily reflect a pediatric hospital-based surveillance population. Suspected dengue cases were defined according to the 2009 WHO dengue case definition, which is routinely applied in Myanmar clinical settings, and disease severity was classified as dengue without warning signs (DWoWS), dengue with warning signs (DWWS), or severe dengue (SD). Patients presented to the hospital within one to seven days after the onset of fever and samples were collected during the patient’s initial hospital visit. Initial DENV diagnostics included rapid antigen testing using SD BIOLINE Dengue NS1 Ag kits (Standard Diagnostic Inc., Suwon, Korea), molecular testing, and serological testing (IgM and IgG in-house ELISA). All serum samples were aliquoted and stored at -80 °C until further use. The archived serum samples were retrospectively analyzed to investigate the potential presence of CHIKV among dengue patients. CHIKV detection was carried out using serological and molecular methods. CHIKV serology was performed on all 267 samples whereas RT-qPCR and downstream analyses were performed on 211 samples due to limited remaining serum volume. The reduction in sample number was due to residual volume only and was unrelated to diagnostic results or clinical characteristics (Fig 1).

### Virus strain and cell lines

The CHIKV S-27 strain (African prototype) was used as the assay antigen for both CHIKV IgM and IgG capture Enzyme-Linked Immunosorbent Assays (ELISA). The virus was propagated at 28°C using C6/36 *Ae. albopictus* mosquito cells grown in Eagle’s Minimum Essential Medium (MEM) supplemented with 10% fetal calf serum (FCS), 50 U/mL penicillin, and 50 µg/mL streptomycin (P/S) [10,16].

### Seroprevalence of DENV and CHIKV antibodies

Detection of anti-DENV NS1 was performed using the SD BIOLINE Dengue Duo combo device (Standard Diagnostic Inc., Korea) following the manufacturer’s instructions. The sera from healthy individuals were used as negative controls to validate the assay performance and determine cutoff thresholds. Detection of anti-DENV IgM and anti-CHIKV IgM was performed on all samples using an in-house IgM capture ELISA system previously described [5,9–11]. For the DENV IgM ELISA, a tetravalent DENV antigen prepared from a mixture of culture fluids infected with the four DENV serotypes was used, together with horseradish peroxidase (HRP)-conjugated anti-flavivirus mouse IgG. For the CHIKV IgM ELISA, CHIKV antigen and HRP-conjugated anti-CHIKV rabbit IgG were used. The IgM P/N ratio was calculated as the OD of the positive control (or sample) divided by the OD of the negative control, and values ≥ 2.0 were considered positive. The CHIKV IgM cutoff (P/N ≥ 2.0) was established through internal validation based on locally collected negative controls and previously published Myanmar serology studies, as cited. Potential cross-reactivity with other co-circulating arboviruses, including flaviviruses, was considered an inherent limitation. Therefore, IgM and IgG results were interpreted in conjunction with molecular findings. Detection of anti-DENV and anti-CHIKV IgG was performed using an in-house indirect IgG ELISA system with DENV and CHIKV antigens respectively [5,9–11]. A positive standard curve was used to determine the IgG titters of DENV and CHIKV, and values ≥ 3,000 were considered positive. [10]. The CHIKV IgG cutoff (≥ 3,000) was determined through internal validation using locally derived negative sera. The DENV IgG ELISA was used to classify primary (< 29,000) and secondary (≥ 29,000) infections, consistent with WHO recommended criteria [5,17].

### CHIKV RNA extraction and genome detection

Molecular testing was performed on 211 samples due to the limited remaining serum volume. Viral RNA was extracted from the patients’ serum samples using the QIAmp Viral RNA Mini kit (cat. No. 52906, Qiagen, Hilden, Germany), following the manufacturer’s instructions. Quantitative RT-qPCR was performed using TaqMan Fast Virus 1-Step Master Mix (cat. No. 4444436, Life Technologies, Carlsbad, CA, USA) with established primers, probe and protocols as previously described [18,19]. The viral genome copies were expressed as log10 genome copies/mL using a standard curve, and a cycle threshold (Ct) value of 37 was used to define positivity. To confirm viral isolation, RNA extracted from the ICF was analyzed by conventional one-step RT-PCR using the Primescript One-Step RT-PCR Kit (Takara Bio Inc., Shiga, Japan) with established primers, and protocols as previously described [5,19].

### Genome sequencing and mutation analysis

To obtain sufficient viral RNA for CHIKV isolation, confirmation, whole-genome sequencing (WGS), and mutation analysis of structural and nonstructural proteins, viruses were isolated by inoculating C6/36 mosquito cells with patient serum. After an initial blind passage, each isolate was passaged twice, ICF supernatant was collected from the second passage (P2) based on cytopathic effects observed after 7 days. CHIKV RNA was extracted from P2 ICF and used for sequencing. Complementary DNA (cDNA) was synthesized using the ReverTra Ace kit (Toyobo, Osaka, Japan), followed by CHIKV multiplex PCR using KOD One PCR Master Mix (Toyobo, Osaka, Japan). The E1 gene was sequenced by Sanger sequencing for genotype confirmation, and WGS was performed to characterize genetic variation and phylogenetic relationships using designed primers (S1 and S2 Tables). The Next-generation sequencing (NGS) was performed on the Illumina MiSeq platform using an amplicon-based approach with a two-pool primer system. Detailed laboratory and bioinformatic workflows are described in the S1 Text. The generated sequences were deposited in NCBI GenBank, and the accession numbers are listed in Table 4 next to the sample numbers.

### Phylogenetic analysis based on the E1 gene

Phylogenetic relationships were inferred from the complete E1 gene sequences of CHIKV, including the 15 isolates obtained in Yangon, Myanmar, and representative reference strains retrieved from GenBank. The dataset comprised 44 nucleotide sequences and 1,320 positions. Sequences were aligned, and evolutionary history was inferred using the Maximum Likelihood (ML) method with the Tamura-Nei substitution model in MEGA version 12. The initial tree for the heuristic search was obtained automatically by applying Neighbor-Join (NJ) and BioNJ algorithms to a matrix of pairwise distances estimated using the Tamura-Nei model, after which the topology with the highest log-likelihood value was selected. A rate-variation model allowing for invariable sites (I) was applied. Bootstrap analysis (1,000 replications) was performed to assess node support.

### Phylogenetic analysis based on the complete genome

Whole-genome phylogenetic analysis was conducted using 55 CHIKV nucleotide sequences (12,056 positions), including the 15 Myanmar isolates and representative sequences of all recognized genotypes obtained from GenBank. Evolutionary history was inferred using the Maximum Likelihood method under the General Time Reversible model with gamma distribution and invariant sites (GTR + G + I), implemented in MEGA version 12. Initial trees were obtained using the Neighbor-Join (NJ) and BioNJ algorithms applied to a matrix of pairwise distances estimated under the GTR + G + I model, and the topology with the highest log-likelihood value was retained. Bootstrap support was calculated with 1,000 replicates to evaluate the reliability of the branches.

### Case definition

For surveillance-based classification in this retrospective dataset, laboratory-confirmed CHIKV infection was defined by detection of viral RNA and/or anti-CHIKV IgM antibodies. However, IgM-based classification was interpreted cautiously, as serological results alone do not establish the timing of infection. DENV infection was defined by NS1 antigen and/or anti-DENV IgM positivity. A co-detection case was defined as laboratory confirmation of both viruses in the same patient [5,10]. This combined definition followed standard diagnostic criteria used in arboviral co-detection studies.

### Statistical analysis

Data were analyzed using IBM SPSS version 21 and GraphPad Prism version 10.4.0. Categorical variables were compared using Chi-square or Fisher’s exact tests, and continuous variables using non-parametric tests, as appropriate. To account for multiple comparisons, p-values were adjusted using the Benjamini-Hochberg false discovery rate (FDR) method (q = 0.10). All analyses were exploratory and descriptive. As this was a retrospective study, no priori power calculation was performed. Given the limited sample sizes in certain subgroups, statistical comparisons were interpreted as exploratory and are presented selectively in the main text, with full results provided in S1 Text.

### Assumption checking

For the Chi-square analyses, the expected cell counts were assessed, and Fisher’s exact test was applied when assumptions were not met. Kruskal-Wallis tests were used for comparison of continuous variables.

## Results

### Demographic characteristics

The demographic characteristics of the study population are summarized in **Table 1**. The study population comprised 267 patients, with slightly more males 141 (52.8%) than females 126 (47.2%), and an overall larger number of children. The participants were categorized into four age groups to maintain consistency with age categorizations used in previous chikungunya epidemiology studies: ≤ 5 years old, 6–15 years old, 16–45 years old, and ≥ 46 years old [20–23]. The age group 6–15 years old had the most participants with 160 (59.9%). The most prevalent type of clinical severity observed was DWWS.

**Table 1:** Demographic and clinical characteristics of the study population.

Variables	Overall (n, %⁺)	Female (n, %⁺⁺)	Male (n, %⁺⁺)
Age groups*			
≤ 5	96 (36.0)	50 (52.1)	46 (47.9)
6-15	160 (59.9)	72 (45.0)	88 (55.0)
16-45	11 (4.1)	4 (36.4)	7 (63.6)
Clinical diagnosis**			
DWoWS	64 (24.0)	33 (51.6)	31 (48.4)
DWWS	188 (70.4)	89 (47.3)	99 (52.7)
SD	15 (5.6)	4 (26.7)	11 (73.3)
**NS1 antigen**			
Positive	168 (62.9)	78 (46.4)	90 (53.6)
Negative	99 (37.1)	48 (48.5)	51 (51.5)
**Laboratory confirmed dengue diagnosis**			
Positive	234 (87.6)	109 (46.6)	125 (53.4)
Negative	33 (12.4)	17 (51.5)	15 (48.5)
**Overall**	267	126 (47.2)	141 (52.8)

⁺ % in total for each variable. ⁺⁺ % within category. * Age groups defined based on the date of illness. ** Clinical diagnosis identified on patient’s initial hospital visit. Proportion of participants characterized by age group, clinical diagnosis, and initial dengue test results.

### Clinicopathological characteristics and serostatus of patients

**Fig 2:**
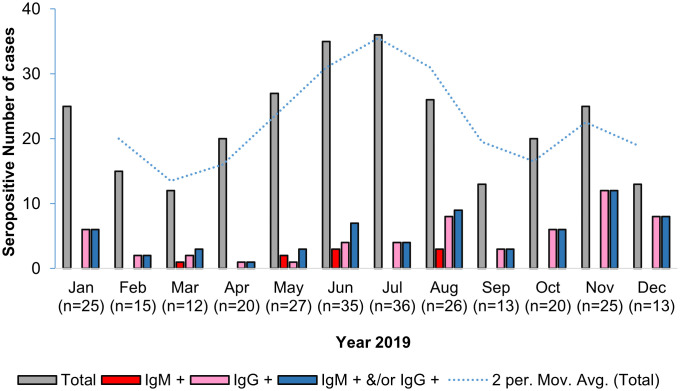
The monthly distribution of CHIKV seropositive cases among dengue-suspected patients.

Of the 267 samples obtained between January and December 2019, 168 (62.9%) were DENV NS1 antigen positive and 99 (37.1%) were DENV NS1 antigen negative. In addition, 196 (73.4%) samples were positive by ELISA IgM, while 193 (72.3%) samples were positive by indirect ELISA IgG (Table 2). To assess infection status, an in-house dengue IgG indirect ELISA test was used to identify samples, the results indicated 61 (22.8%) as primary infection, 173 (64.8%) as secondary infection, 20 (7.5%) as past-Flavivirus infection, and 13 (4.9%) as non-dengue infection. Subsequent anti-CHIKV in-house testing revealed that 9 of 267 (3.4%) samples were CHIKV IgM positive: 57/267 (21.3%) were CHIKV IgG positive, corresponding to an overall anti-CHIKV seropositive of 66/267 (24.7%) (Table 2). Given the small number of CHIKV IgM-positive cases, subgroup patterns are presented descriptively and should be interpreted with caution. Most samples were collected between May and July, with a peak in July. CHIKV IgM-positive cases were observed predominantly during this period whereas IgG positivity was more frequent in January, November, and December, with no IgM detected (Fig 2).

**Table 2:** Descriptive clinical presentation of patients with detectable anti-CHIKV antibodies among dengue-suspected patients in Yangon, Myanmar.

Variables	Overall n (%)⁺	IgM positive n (%)⁺⁺, (1)	IgG positive n (%)⁺⁺, (2)	IgM &/or IgG positive n (%)⁺⁺,(3)
**Genders**				
Female	126 (47.2)	7 (5.6)	25 (19.8)	32 (25.4)
Male	141 (52.8)	2 (1.4)	32 (22.7)	34 (24.1)
Age groups*				
≤ 5	96 (36.0)	5 (5.2)	19 (19.8)	24 (25)
6-15	160 (59.9)	3 (1.9)	37 (23.1)	40 (25.0)
16-45	11 (4.1)	1 (9.1)	1 (9.1)	2 (18.2)
Clinical diagnosis**				
DWoWS	64 (24.0)	3 (4.7)	12 (18.8)	15 (23.4)
DWWS	188 (70.4)	6 (3.2)	42 (22.3)	48 (25.5)
SD	15 (5.6)		3 (20.0)	3 (20.0)
**NS1 antigen**				
Positive	168 (62.9)	5 (3.0)	40 (23.8)	45 (26.8)
Negative	99 (37.1)	4 (4.0)	17 (17.2)	21 (21.2)
**DENV IgM**				
Positive	196 (73.4)	7 (3.6)	44 (22.4)	51 (26.0)
Negative	71 (26.6)	2 (2.8)	13 (18.3)	15 (21.1)
**DENV IgG**				
Positive	193 (72.3)	7 (3.6)	41 (21.2)	48 (24.9)
Negative	74 (27.7)	2 (2.7)	16 (21.6)	18 (23.3)
**Overall**	267 (100)	9 (3.4)	57 (21.3)	66 (24.7)

⁺ % in total for each variable. ⁺⁺ % within category. * Age groups defined based on the date of illness. ** Clinical diagnosis identified on patient’s initial hospital visit. Proportion of patients with only IgM positive (1), only IgG positive (2), and IgM positive and/or IgG positive (3) compared by genders, age groups, and clinical diagnosis and DENV clinical status.

Clinical and laboratory characteristics were compared among patients with DENV infection, CHIKV infection, and DENV-CHIKV co-detection. Statistically significant differences were observed across the three groups for NS1 antigen status, joint pain, and hepatomegaly (S3 Table). In addition, differences in days of fever and platelet count were observed among the infection groups.

### Molecular and clinical features of CHIKV RNA-positive patients

**Fig 3:**
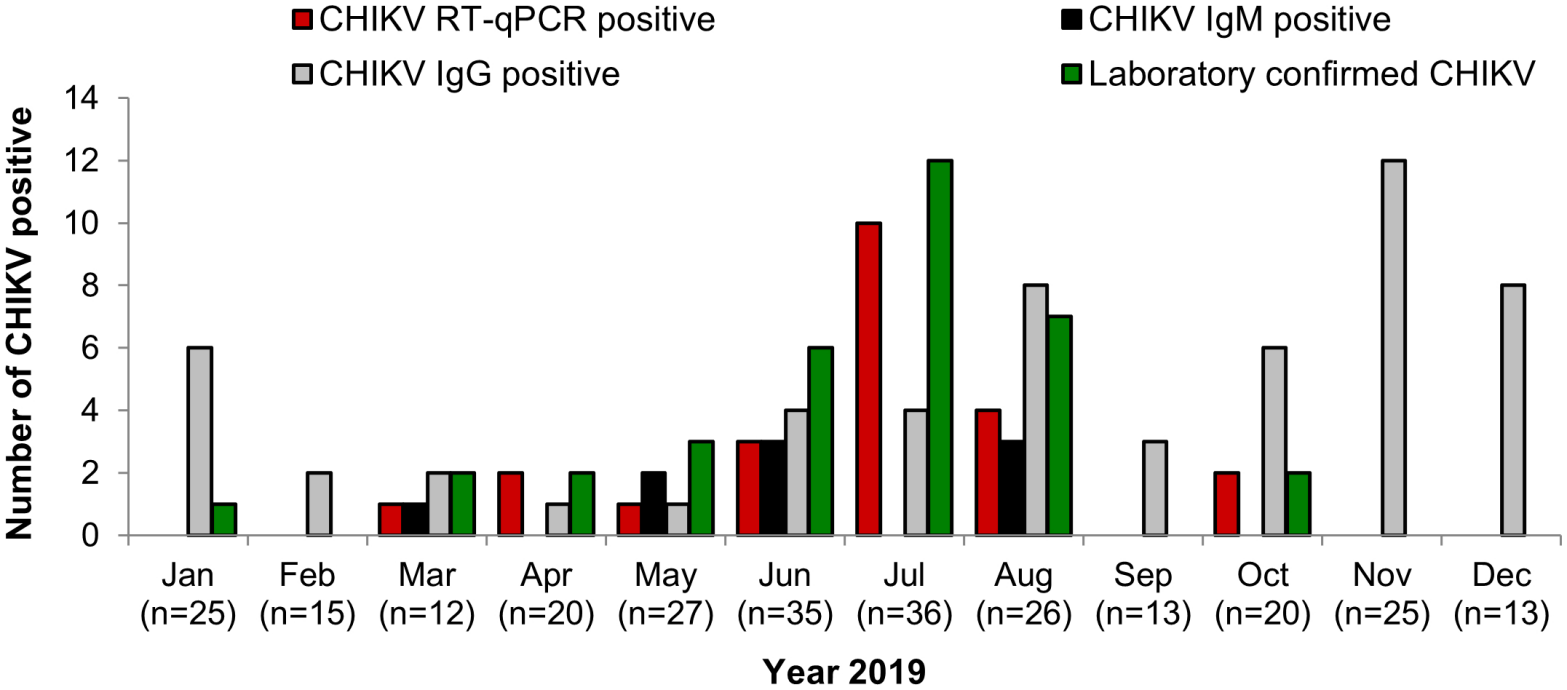
The Monthly distribution of CHIKV diagnostic outcomes among dengue-suspected patients.

**Fig 4:**
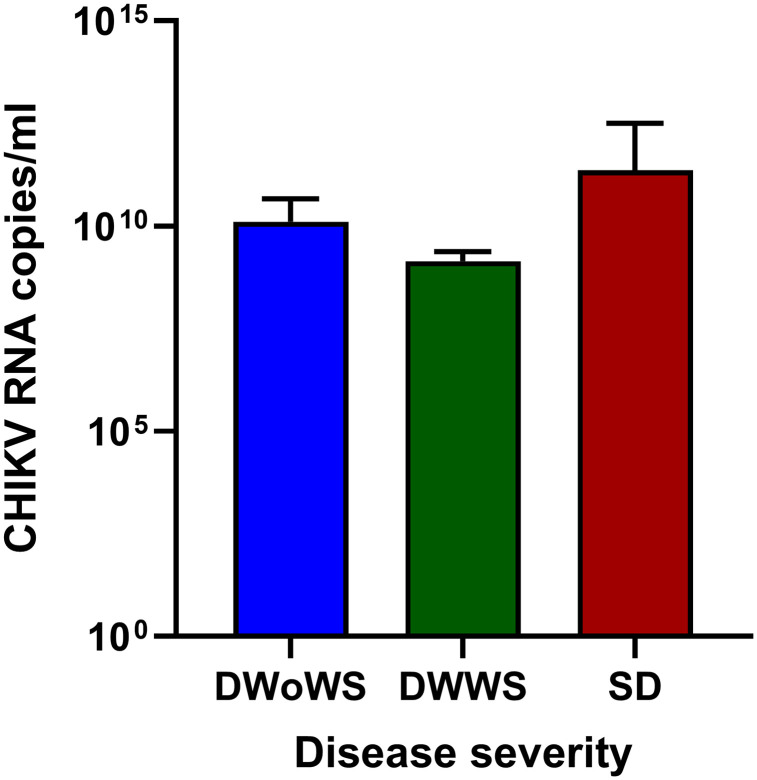
The Distribution of CHIKV RNA viral Load (log₁₀ copies/mL) in dengue-suspected patients by clinical severity. DWoWS: Dengue without warning signs, DWWS: Dengue with warning signs, SD: Severe dengue.

Molecular analysis was performed on 211 dengue-suspected samples. CHIKV RNA was detected in 23 of 211 patients (10.9%), with viral loads ranging from 4x10^5^ to 4x10^11^copies/mL. The detection rate was highest among children aged 6–15 years (17/125; 13.6%), followed by ≤5 years (6/75; 8%), with no RNA detected in patients aged ≥16 years (0/11). Males showed slightly higher detection rates (13/108; 12.0%) than females (10/103; 9.7%) (Table 3). CHIKV RNA positivity was associated with DENV NS1 antigen status (23.2% in NS1-negative vs 0% in NS1-positive; Fisher’s exact P-value < 0.001). There were no significant differences observed by sex, age groups and clinical diagnosis after correction for multiple testing. Detailed symptom-based and extended statistical comparisons are provided in supplementary (S3 and S6 Tables). CHIKV RNA-positive cases, including isolates, were detected between March and October, with a cluster from June to August. The highest number of PCR positive cases occurred in July (Fig 3). CHIKV RNA was detected across all clinical severity categories with 6/48 (12.5%) DWoWS, 15/153 (9.8%) DWWS, and 2/10 (20.0%) SD. Although higher viral loads (>10¹⁰ copies/mL) were observed in severe dengue cases, these observations are based on small numbers and should be interpreted cautiously (Fig 4).

**Table 3:** Molecular detection of CHIKV infection among dengue suspected patients in 2019 Yangon, Myanmar.

Variables	Patients n (%)	CHIKV RNA positive n (%)	CHIKV RNA negative n (%)	P-value
**Gender**				0.662
Female	103 (48.8)	10 (9.7)	93 (90.3)	
Male	108 (51.2)	13 (12.0)	95 (88.0)	
Age groups*				0.577
≤ 5	75 (35.6)	6 (8.0)	69 (92.0)	
6-15	125 (59.2)	17 (13.6)	108 (86.4)	
16-45	11 (5.2)	0	11 (100)	
Clinical diagnosis**				0.231
DWoWS	48 (22.8)	6 (12.5)	42 (87.5)	
DWWS	153 (72.5)	15 (9.8)	138 (90.2)	
SD	10 (4.7)	2 (20.0)	8 (80.0)	
**NS1 Antigen**				<0.001
Positive	112 (53.1)	0	112 (100)	
Negative	99 (46.9)	23 (23.2)	76 (76.8)	
**Overall**	211 (100)	23 (10.9)	188 (89.1)	

⁺ % in total for each variable. ⁺⁺ % within category. * Age groups defined based on the date of illness. ** Clinical diagnosis identified on patient’s initial hospital visit. Adjusted p-values were calculated using the Benjamini-Hochberg false discovery rate (FDR) method across variable blocks.

**Fig 5:**
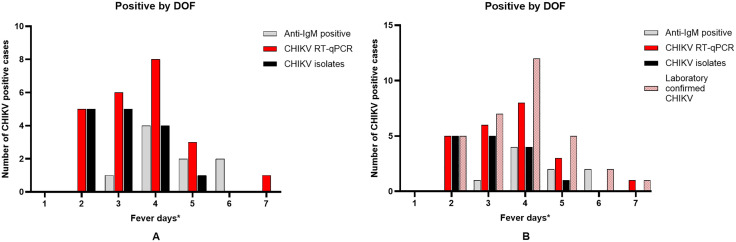
The distribution of CHIKV cases by days of fever. *Number of days after onset of fever. CHIKV IgM-positive, RT-qPCR-positive, virus isolation (A), and laboratory confirmed CHIKV (B) positive cases by days of fever at the time of sample collection.

Among the 23 CHIKV RT-qPCR-positive samples, 2 samples had previously tested positive for DENV serotype 2 (DENV-2) by RT-qPCR. All CHIKV RNA-positive patients were DENV NS1-negative. Regarding DENV infection status, among the 23 CHIKV RNA-positive patients, 1 was classified as a primary infection, 9 as secondary infections, 10 as past-flavivirus infections, and 3 as non-dengue cases. Fever duration among CHIKV RT-qPCR-positive and virus isolation-positive patients peaked on day 4, whereas IgM-positive cases showed peaks on days 4 and 6 (Fig 5).

### CHIKV virus isolation and associated clinical characteristics

CHIKV virus isolation was performed on the subset of 211 samples, resulting in 15 successful isolates 7.1% (15/211). Isolation was successful in 11/108 males (10.2%) and 4/103 females (3.8%). The highest isolation rate was observed in children aged 6–15 years (11/125; 8.8%), followed by ≤5 years (4/75; 5.3%). Of the 15 CHIKV isolates confirmed by conventional PCR, 2 were also positive for DENV-2 RNA by RT-qPCR, although DENV was not isolated. Isolates were obtained from 4 of 48 patients with DWoWS (8.3%) and 11 of 153 with DWWS (7.2%) with none recovered from patients with SD. Dengue serological profiles showed that isolates were obtained from 1 primary dengue infection, 5 secondary infections, 6 past flavivirus infections, and 3 non-dengue (Table 4).

**Table 4:** Characterization of CHIKV isolates identified among Dengue-Suspected patients in Yangon, Myanmar during 2019.

GenBank Accession no	ID	Age (Year)	Days of fever	Clinical severity	CHIKV IgM P/N ratio	CHIKV IgG titer	DENV IgM P/N ratio	DENV IgG titer	DENV infection type	CHIKV copies/ml	DENV copies/ml	DENV RNA
PV664498	082	11	2	DWoWS	0.8	815	1.1	20,201	Past flavi-infection	3.74x10^6^		ND
PV683444	150	12	3	DWoWS	0.7	493	1.6	25,842	Past flavi-infection	9.35 x10^6^		ND
PV683445	151	8	2	DWWS	1.7	1,040	1.1	66,840	Secondary	1.26 x10^7^		ND
PV683446	185	4	3	DWWS	1.2	1,835	0.5	180	Non-dengue	2.73 x10^9^		ND
PV683447	188	4.5	3	DWWS	1.2	1,089	0.3	5,656	Past flavi-infection	1.48 x10^9^		ND
PV683448	195	4.5	2	DWoWS	1	701	0.3	134	Primary	7.70 x10^10^	3.88 x10^**4**^	DENV-2
PV683449	199	9	4	DWWS	0.8	1,015	0.4	1,182	Non-dengue	9.22 x10^7^		ND
PV683450	212	8	5	DWWS	1.2	807	0.3	5,172	Past flavi-infection	2.53 x10^9^		ND
PV683451	213	8	4	DWWS	1.2	982	0.6	15,738	Secondary	5.55 x10^9^	1.09 x10^**5**^	DENV-2
PV683452	230	11	3	DWoWS	0.6	1,527	3	20,089	Secondary	4.10 x10^7^		ND
PV683453	232	8	3	DWWS	0.6	486	0.7	3,306	Past flavi-infection	1.29 x10^8^		ND
PV683454	240	4	2	DWWS	1.2	886	0.3	844	Non-dengue	2.34 x10^8^		ND
PV683455	259	8	4	DWWS	0.5	708	0.8	38,127	Secondary	3.67 x10^9^		ND
PV683456	262	12	4	DWWS	0.4	1,148	0.5	44,462	Secondary	9.30 x10^8^		ND
PV683457	301	6	2	DWWS	1.5	486	0.9	23,769	Past flavi-infection	3.61 x10^9^		ND

Cut off value of IgM-positive = **≥** 2.0; IgG-positive = **≥** 3000. P/N: positive-to-negative ratio. ND: not detected.

### Phylogenetic analysis and genomic characterization

**Fig 6:**
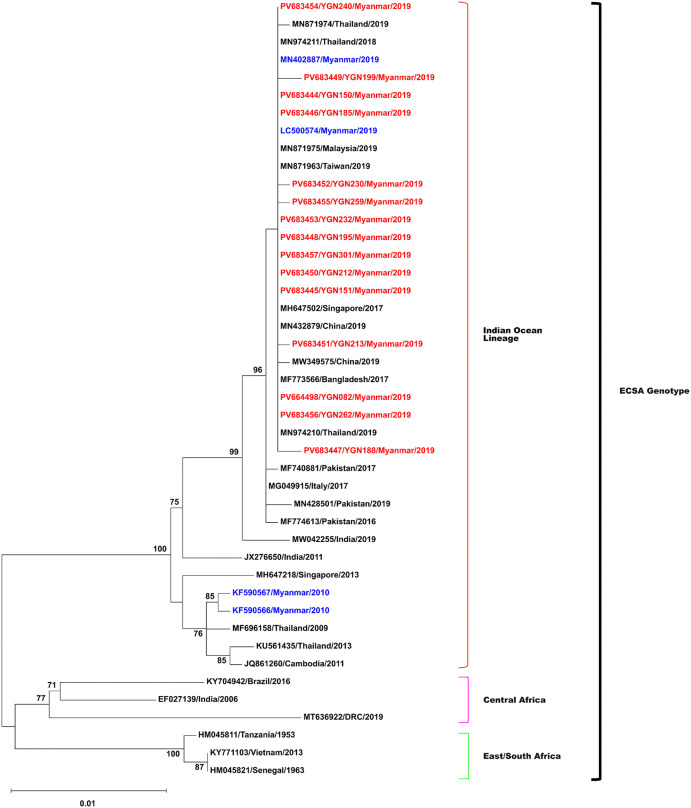
Maximum Likelihood phylogeny of CHIKV E1 gene sequences showing Myanmar isolates (red) within the Indian Ocean Lineage of the ECSA genotype. Previous Myanmar isolates are in blue.

**Fig 7:**
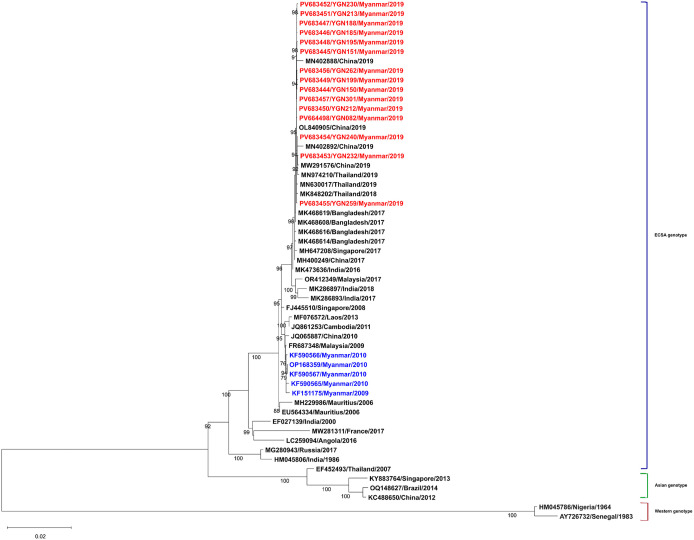
Maximum Likelihood phylogeny based on CHIKV whole genomes showing Myanmar isolates (red) clustering within the ECSA genotype; previous Myanmar isolates are in blue.

To characterize the genomic features of CHIKV isolates in this study, we sequenced the 15 isolates for the E1 gene and analyzed complete genome sequences in comparison with global CHIKV strains. Phylogenetic trees based on the E1 gene (Fig 6) and complete genome sequences (Fig 7) showed that all isolates belonged to the East/Central/South African (ECSA) genotype, Indian Ocean Lineage (IOL), and clustered with strains from China (MN432879), Thailand (MN974210), Bangladesh (MF773566), and Mandalay, Myanmar (MN402887). The isolates were part of multiple clades within the ECSA-IOL lineage. Whole-genome sequencing produced consensus sequences with a median coverage exceeding 1,000× and more than 98% genome completeness, consistent with the expected CHIKV genome length (~11.7 kb). A total of 33 non-synonymous amino acid substitutions were identified across non-structural (20) and structural (13) proteins (S4 Table). Among these, 22 substitutions have been previously reported in ECSA-IOL strains, while 11 substitutions were not documented in the Myanmar reference sequences included in this analysis (S5 Table). The substitutions NSP2-E145D, NSP4-S55N, E2-V264A, and E1-K211E have been previously reported in regional ECSA-IOL strains. Seventeen substitutions were present in all 15 isolates and were distributed across the genome while 12 substitutions were detected in single isolates. No clear association between mutation patterns and disease severity was observed in this dataset (S6 Table). Bayesian analysis comparing isolates from 2009, 2010, and 2019 (S1 Fig) showed genetic differences between sampling years. Mutation pattern comparisons (S2 Fig) demonstrated variation in substitution profiles across years. BLAST comparison with global sequences confirmed clustering of the Yangon isolates with strains from Thailand, China, Bangladesh, and Mandalay (S3 Fig).

## Discussion

CHIKV and DENV are co-endemic in Myanmar and share overlapping symptoms, yet CHIKV surveillance remains limited [11]. The Yangon region records the highest number of dengue cases and a high prevalence of *Aedes* mosquitoes, as observed during the 2019 outbreak [11,24,25]. Following the 2019 dengue outbreak in Yangon, clinical samples initially collected for DENV testing [14] were retrospectively re-examined to investigate CHIKV circulation. CHIKV activity was also reported in neighboring countries during the same period [7,10,11,26]. Our serological findings demonstrated positive results for CHIKV IgM in 3.4% and IgG in 21.3% of dengue-suspected patients. The IgM seropositivity was lower, while IgG seropositivity was comparable to reports from Indonesia (IgM: 13.3%, IgG: 18.5%) and Vietnam in 2019 (IgM: 15.4%, IgG: 20.9%) [27,28], and aligned with findings from Mandalay during a DENV outbreak [5,9]. Taken together, these findings are consistent with prior CHIKV exposure in a co-endemic setting [5,9–11,27–29]. Among DENV NS1-negative samples, 4% were CHIKV IgM positive, consistent with WHO guidance to evaluate dengue-negative febrile cases in endemic regions. Notably, all RNA-positive cases were seronegative for both IgM and IgG, which may reflect sampling during the early phase of infection prior to IgM seroconversion, when antibodies are not yet detectable. In addition, the sensitivity limitations of ELISA-based assays may contribute to underdetection of low antibody levels. Nonetheless, because IgM ELISA assays can show cross-reaction between DENV and CHIKV, the observed CHIKV IgM positivity may reflect serological overlap common in co-endemic regions, where overlapping immune responses may further complicate interpretation. CHIKV RNA was detected in 10.9% (23/211) of tested dengue-suspected patients, similar to the 11% reported in Mandalay in 2019 [11]. During the same period, CHIKV RNA was also reported in neighboring countries and regions, including China (53.8%), Vietnam (33.3%), Thailand (4.9%), India (6.3%) and Mandalay with cases identified among healthy individuals, dengue-negative patients, and dengue-suspected cases [5,10,28,30,31]. These observations are consistent with regional CHIKV circulation during 2019. Among CHIKV RT-qPCR positive cases, two were also DENV-2 positive, representing molecular co-detection. Similar findings were reported in travelers returning from Myanmar to China and in studies describing concurrent detection of DENV and CHIKV in Brazil [32,33]. Five CHIKV RNA-positive patients were classified as secondary DENV infections, which may reflect sequential infections or serological cross-reactivity. These findings illustrate the diagnostic complexity of arboviral co-circulation. The descriptive comparisons across dengue infection, chikungunya infection, and DENV-CHIKV co-detection groups identified differences in the NS1 test, joint pain and hepatomegaly. Similar findings were also observed in infections involving the co-detection of DENV serotype 2 and CHIKV [33]. Nonetheless, given the small and uneven group sizes in our study, our comparisons should be interpreted cautiously. All the CHIKV RNA-positive cases occurred in DENV NS1-negative individuals. A multiplex study detected CHIKV RNA in dengue-suspected patients who were exclusively DENV-NS1 negative [34]. A study conducted on clinically diagnosed dengue patients in the Philippines, detected CHIKV RNA in 10% of the samples [35]. This pattern is consistent with detection of CHIKV RNA before measurable antibody responses, as reported in other settings [30]. These observations highlight the complementary role of molecular testing during early infection in co-endemic settings. The detection of CHIKV among dengue-suspected patients in this study aligns with reports from multiple countries such as India, Laos and Sri Lanka where concurrent circulation has been documented during dengue outbreaks [36–40]. The CHIKV RNA detection peaked mid-year, corresponding to the monsoon season. Temporal clustering likely reflects increased healthcare presentation during this period and may have introduced sampling bias. The virus was successfully isolated in 15/211 patients (7.1%), predominantly from children aged 6–15 years. Although some isolates originated from samples positive for DENV RNA, DENV was not isolated. Arbovirus surveillance in Mexico also detected and isolated CHIKV in patients with and without DF [41]. These findings confirm CHIKV viability during the outbreak but do not imply viral interference. Isolation was most successful during the early febrile phase (days 2–4), consistent with known virological dynamics. Mutation analysis revealed that the CHIKV isolates in this study belonged to the Indian Ocean subclade of the East/Central/South African genotype. In this study, most of the mutations (17/33) occurred in all sequenced isolates, and were shared across both non-structural and structural proteins, including those involved in genome replication (NSP1-NSP4), RNA capping, host cell entry (E1, E2), and virion assembly (capsid) [42–52]. Most of the known mutations found in all our isolates were also reported in the ECSA genotype of CHIKV isolates from post-2015 outbreaks in South and West Asia, including China, India, Thailand, Pakistan, Bangladesh, Malaysia and Myanmar [7,32,53–55]. These findings are consistent with circulation of related ECSA-IOL strains in the region. The E1 gene of our isolates did not harbor the E1-A226V mutation, which has been previously reported in ECSA-IOL strains, including Myanmar 2010 CHIKV isolates [32,56]. Instead, our isolates retained the E1-226A residue, a pattern also described in ECSA-IOL strains circulating in different epidemiological settings [56]. The isolates also harbored the E1-K211E and E2-V264A mutations, which, together with E1-226A, have been reported in ECSA-IOL strains circulating across Asia and Africa, including China, Malaysia, India, and Kenya [53,57]. Thailand and Malaysia have observed similar E1 mutations in their isolates [26,31,55]. No functional assays were performed in this study, and no inference regarding viral fitness, transmission efficiency, or vector adaptation can be made from the present data. Eleven substitutions were observed only among the Yangon isolates included in this study. Their biological relevance remains unknown and warrants further investigation [58,59]. We also identified 12 mutations that were found in only one isolate each as observed in other endemic regions [60]. The mutations occurred across various viral proteins, including NSP1–4, E2, and E1 and were observed across a range of viral load values. Notably, two samples (PV683448 and PV683451) were also DENV-2 positive. Their biological relevance cannot be determined from this dataset [7]. There was no significant association between mutations and disease severity. Our findings are consistent with reports from neighboring South and Southeast Asian countries, including India, Bangladesh, Thailand, Malaysia, and China, where ECSA-IOL CHIKV strains carrying E1-K211E and E2-V264A have been reported since 2016 [26,55,57,61–63]. The absence of the E1-A226V mutation and the presence of E1-226A, E1-K211E, and E2-V264A observed in our study have been described in India and Bangladesh [57,61,64]. Bayesian and mutation analyses based on the E1 gene identified mutations present in both 2010 and 2019, including additional substitutions in the latter. Similar observations have been reported in China, Thailand, and Bangladesh during the same period [62,65,66]. As a retrospective analysis, this study has inherent limitations. Serological results may have been affected by cross-reactivity with other arboviruses, and IgM positivity may reflect recent or waning infection rather than active disease. Molecular detection and genome sequencing were limited by residual sample volume, long-term storage, and freeze-thaw cycles, potentially reducing sensitivity and viral viability. The hospital-based, pediatric-weighted sample limits generalizability, and the absence of RNA-positive adults likely reflects small adult numbers rather than true absence of infection. Archived samples and lack of follow-up restricted clinical outcome assessment. The small number of isolates (n = 15) may not capture full viral diversity, and reference-based genome assembly may introduce bias despite adequate coverage. Functional effects of detected mutations were not assessed, and genomic findings are descriptive. Nonetheless, the study confirms CHIKV occurrence among dengue-suspected patients in Yangon, informing differential diagnosis during outbreaks.

## Conclusion

In summary, this study documents CHIKV detection, isolation, and genomic characterization among dengue-suspected patients in Yangon during the 2019 outbreak. While not estimating population burden, the findings highlight the importance of considering CHIKV in differential diagnosis during dengue outbreaks. Continued molecular surveillance remains a relevant public health consideration of arboviral infections in co-endemic settings.

## Supporting information

S1 Text Whole genome sequencing Methodology. The sequencing methodology of whole-genome and E1 gene analysis with the detailed description of the primer design, cDNA synthesis, multiplex PCR, and Illumina sequencing workflow for the CHIKV isolates including the library preparation, bioinformatic analysis (MAFFT, BWA, SAMtools, MEGA 12), and consensus sequence generation.
(DOCX)

S1 Table CHIKV primers used for E1 gene. Primer sequences employed for CHIKV E1 gene amplification in real-time RT-qPCR and conventional RT-PCR.
(DOCX)

S2 Table CHIKV primer pairs (forward and reverse odd and even numbered primer pairs) used for Whole genome sequencing. The list of the primer sequences used for the amplicon-based whole-genome amplification (two-pool system: odd primer and even primer sets).
(DOCX)

S3 Table Descriptive comparison of demographic, clinical and laboratory profiles of dengue, chikungunya, and co-detection cases in dengue-suspected patients in Yangon Myanmar. Distribution of categorical demographic and clinical variables across infection groups. Comparisons were exploratory.
(DOCX)

S4 Table Alignment of amino acid sequences of CHIKV isolates in this study in comparison with Myanmar reference strains. (A) Nonstructural proteins and (B) Structural protein. (A) Shows the non-structural proteins amino acid’s differences; (B) Shows the structural proteins amino acid’s differences. The dashes indicate the conserved residues.
(DOCX)

S5 Table (A) Distribution of CHIKV Mutations Across Non-Structural and Structural Proteins from Whole Genome Sequences of Isolates in Dengue-Suspected Patients, Yangon, Myanmar (2019). **(B) Mutation Analysis of CHIKV isolates positives from dengue-suspected patients in Yangon, Myanmar 2019.** A comprehensive list of amino-acid substitutions identified in the 15 CHIKV isolates, including functional insights, biological impacts, and supporting literature references.
(DOCX)

S6 Table Association between CHIKV amino-acid mutations and disease severity. The Fisher’s exact test results for associations between amino-acid mutations and disease severity (DWoWS vs DWWS). The adjusted p-values were computed using the Benjamini-Hochberg method.
(DOCX)

S1 Fig Bayesian phylogenetic tree of Chikungunya virus E1 gene sequences from Myanmar (2009–2019). A time-scaled Bayesian phylogenetic tree generated in BEAST v1.10.4 illustrating the temporal clustering of 2009, 2010, and 2019 Myanmar isolates within the ECSA lineage (red: study isolates).
(TIF)

S2 Fig Heatmap of amino acid mutations in the E1 gene of Chikungunya virus isolates from Myanmar (2009–2019). The heatmap summarizing amino-acid substitutions across Myanmar isolates highlighting conserved and variable sites within the ECSA genotype (red: study 15 isolates).
(TIF)

S3 Fig Global maximum-likelihood phylogenetic tree of Chikungunya virus E1 gene sequences. The Maximum-likelihood tree (IQ-TREE 2) constructed from 461 E1 gene sequences, including 15 Myanmar isolates from this study and 446 global strains. Myanmar isolates are shown in red (study isolates) and blue (previous Myanmar strains).
(TIF)
