# Supplementary material for: Chikungunya virus in dengue-suspected patients: Molecular evidence from the 2019 outbreak in Yangon, Myanmar
Source: PLoS Negl Trop Dis. 2026 May 4;20(5):e0014258. doi: 10.1371/journal.pntd.0014258 (PMC13138656; doi:10.1371/journal.pntd.0014258)
Supplement: S1 Text — The sequencing methodology of whole-genome and E1 gene analysis with the detailed description of the primer design, cDNA synthesis, multiplex PCR, and Illumina sequencing workflow for the CHIKV isolates including the library preparation, bioinformatic analysis (MAFFT, BWA, SAMtools, MEGA 12), and consensus sequence generation. (DOCX) [file pntd.0014258.s001.docx]

**Supplementary**

**Appendix 1. Sequencing Methodology**

**1.1. Primer Design for Amplicon Sequencing**

FASTA sequence data for the CHIKV whole genome sequencing were retrieved from the NCBI database to create two multiple-sequence datasets. Sequences were aligned using MAFFT (v7.520), and consensus sequences were generated with SnapGene (v8.0.1). Based on the consensus, primers were designed using Primer3 to amplify a 294 bp region of the E1 gene (Supplementary Table 1) and ~300 bp overlapping amplicons spanning the full genome (Supplementary Table 2).

**1.2. cDNA Synthesis**

To obtain cDNA from CHIKV RNA-positive isolates, reverse transcription was performed using the ReverTra Ace kit (Toyobo, Osaka, Japan), following the manufacturer’s instructions.

**1.3. Whole Genome Multiplex PCR**

The whole genome amplification was performed using an amplicon-based strategy with a two-pool primer system, in which Pool 1 contained odd-numbered primer pairs and Pool 2 contained even-numbered primer pairs to minimize amplicon overlap.

-Primers were diluted to 50 µM in nuclease-free water. For each sample, two 50 µL PCR reactions were prepared using KOD One PCR Master Mix:

- **Pool 1**: 25 µL Master Mix, 16.2 µL water, 7.8 µL Primer Pool 1 (final concentration 0.3 µM), 1 µL cDNA
- **Pool 2**: 25 µL Master Mix, 16.8 µL water, 7.2 µL Primer Pool 2 (final concentration 0.3 µM), 1 µL cDNA

PCR cycling conditions:
98 °C for 10 s → 50 °C for 5 s → 68 °C for 10 s, for 30 cycles, followed by a 4 °C hold.

The amplicons from both reactions were pooled (10 µL total per sample) and purified using AMPure XP beads (Beckman Coulter, USA). DNA concentration was quantified using a Qubit 2.0 Fluorometer (Invitrogen, USA).

**1.4. Illumina Sequencing and Analysis**

DNA libraries were prepared using the QIAseq FX DNA Library Kit (Qiagen, Germany), following the manufacturer’s protocol. Quality control was performed using Qubit and Bioanalyzer High-Sensitivity DNA Analysis (Agilent, USA).

Sequencing was carried out on the Illumina MiSeq platform using a 2×150 bp paired-end MiSeq reagent kit v2. Raw FASTQ files were assessed using FastQC (v0.11.9) and FASTP (v0.23.4). Filtered reads were aligned to the CHIKV reference genome (Mandalay 2019 strain, GenBank: OP168359) using BWA (v0.7.17-r1188), and consensus sequences were generated with SAMtools (v1.20).

**1.5. Phylogenetic and Mutation Analysis**

Consensus sequences from the 15 CHIKV isolates were aligned with previously reported sequences using MAFFT (v7.520). Phylogenetic trees were constructed in MEGA12 using the maximum likelihood method with 1,000 bootstrap replicates. Figures were refined using Inkscape (v1.2.1). Amino acid comparisons and mutation analysis were conducted in Jalview (v2.11.4.0), using the 2010 CHIKV Myanmar strain (GenBank: KF590567) as the reference.

**Supplementary figures titles and legends**

**S1 Figure. Bayesian phylogenetic tree of Chikungunya virus E1 gene sequences from Myanmar (2009-2019).**

A time-scaled Bayesian phylogenetic tree was generated in BEAST v1.10.4 based on E1 gene sequences from Myanmar isolates collected in 2009, 2010, and 2019. The tree illustrates temporal clustering and evolutionary relationships among Myanmar strains within the ECSA lineage. (Red: Study 15 isolates)

**S2 Figure. Heatmap of amino acid mutations in the E1 gene of Chikungunya virus isolates from Myanmar (2009-2019).**

The heatmap summarizes amino acid substitutions identified in the E1 gene of Myanmar isolates from 2009, 2010, and 2019. Mutation patterns are visualized across strains, highlighting conserved and variable positions within the ECSA genotype. (Red: Study 15 isolates)

**S3 Figure. Global maximum-likelihood phylogenetic tree of Chikungunya virus E1 gene sequences.**

A maximum-likelihood phylogenetic tree was constructed using IQ-TREE2 based on the E1 gene sequences of 461 strains, including the 15 isolates from this study and 446 global strains retrieved from NCBI. Myanmar isolates are highlighted (Red: study isolates, Blue: previous Myanmar strains) to show their clustering within the ECSA lineage and their relationship to previously reported Asian and Indian Ocean lineage strains.
