# Supplementary material for: Chikungunya virus in dengue-suspected patients: Molecular evidence from the 2019 outbreak in Yangon, Myanmar
Source: PLoS Negl Trop Dis. 2026 May 4;20(5):e0014258. doi: 10.1371/journal.pntd.0014258 (PMC13138656; doi:10.1371/journal.pntd.0014258)
Supplement: S1 Table — Primer sequences employed for CHIKV E1 gene amplification in real-time RT-qPCR and conventional RT-PCR. (DOCX) [file pntd.0014258.s002.docx]

**S1 Table:** CHIKV primers used for E1 gene.

| **Primer** | **Sequence (5′ → 3′)** |
| --- | --- |
| **Real-time RT-qPCR** | |
| CHIKV 874 874-894 -F | AAAGGGCAAACTCAGCTTCAC |
| CHIKV 961 961-942 -R | GCCTGGGCTCATCGTTATTC |
| CHIKV 899-FAM§ 899-923 | CGCTGTGATACAGTGGTTTCGTGTG-BHQ-1 |
| **Conventional RT-PCR and Sequencing** | |
| Forward primer - P3F | TGCGGTATACCTGTGGAACG |
| Reverse primer - P3R | TTTGTACTCGCACGTGATGT |
| Forward primer - P4F | AACAGTGATCCCGAACACGG |
| Reverse primer - P4R | ATTGTCCTGGTCTTCCTGCGAT |
| Forward primer - P5F | CATCAGCGTACAGGGCTCAT |
| Reverse primer - P5R | GACTTGTACGCGGAATTCGG |
| Forward primer - P6F | ATTGGCTAAAAGAACGCGGG |
| Reverse primer - P6R | TAGTGCCTGCTGAACGACAC |
