# Supplementary material for: Chikungunya virus in dengue-suspected patients: Molecular evidence from the 2019 outbreak in Yangon, Myanmar
Source: PLoS Negl Trop Dis. 2026 May 4;20(5):e0014258. doi: 10.1371/journal.pntd.0014258 (PMC13138656; doi:10.1371/journal.pntd.0014258)
Supplement: S2 Table — The list of the primer sequences used for the amplicon-based whole-genome amplification (two-pool system: odd primer and even primer sets). (DOCX) [file pntd.0014258.s003.docx]

**S2 Table**: **CHIKV primer pairs (forward and reverse odd and even numbered primer pairs) used for Whole genome sequencing.**

| **Name** | **Sequence** |
| --- | --- |
| CHIKV-F1 | GACACACGTAGCCTACCAGT |
| CHIKV-R1 | GCATTGGCATGGTCATTCGG |
| CHIKV-F2 | TGGAACCAAGGCAGGTCACA |
| CHIKV-R2 | TGCTCATCTGCCCAGTTTGT |
| CHIKV-F3 | GTGCCTACCCCTCCTACTCGTA |
| CHIKV-R3 | GTTGCGTTCTGCCGTTAACC |
| CHIKV-F4 | GGGCTGAACCAGAGAATAGTC |
| CHIKV-R4 | TCTATTATTCCTGCGCCCGCAT |
| CHIKV-F5 | TGGAACAGCTTGAGGACAGA |
| CHIKV-R5 | TTTTGTATGGGCAGGCAGGGA |
| CHIKV-F6 | TATGAAGGGCTAAAAATCCGC |
| CHIKV-R6 | TAGTGCGCATTTTGCCTTCG |
| CHIKV-F7 | ATTGTGTCATCGTTGCATTAGC |
| CHIKV-R7 | TCTGAGACCACTGCCTATCAC |
| CHIKV-F8 | AACAGCGGGGATAAAACTAAC |
| CHIKV-R8 | GCTGGCAGACCCAACTCTAG |
| CHIKV-F9 | GAGCGGACTACACATACAAC |
| CHIKV-R9 | TGTTCTTAAAGGACTCCGGCCAT |
| CHIKV-F10 | CAAGGCAGTATATGCAAATCG |
| CHIKV-R10 | TCCACAGCCGTCTGATGAAAC |
| CHIKV-F11 | CATATCTAGAAGGGACCCGT |
| CHIKV-R11 | CCCGTCTTCTAGTGCTGGTT |
| CHIKV-F12 | TGCTGACGCCCCAGCCCTAG |
| CHIKV-R12 | GACCAGTGTCCGACGAGAAT |
| CHIKV-F13 | GACAGGGCAGGTGGGTATATGC |
| CHIKV-R13 | GATCGGAGGCGAGTACACAG |
| CHIKV-F14 | TCGGACCACATATCCGGCGCTA |
| CHIKV-R14 | GGGCTGGCAGCAAATTCTTC |
| CHIKV-F15 | CATGCAACCAAGAATACTGG |
| CHIKV-R15 | CGGTAGGTGACAGCTGGAAA |
| CHIKV-F16 | AGAGGCTGCTTTCGGAGAGA |
| CHIKV-R16 | TGGCCATGGACATTACCACA |
| CHIKV-F17 | GAAGTGCAGGGTATATCAGT |
| CHIKV-R17 | ACCTTCGTGCTTGACTTCGAA |
| CHIKV-F18 | AAATCGAAAATGATTGTGATGC |
| CHIKV-R18 | GAACGTGGTGTTTGCCAGCA |
| CHIKV-F19 | GCCATCCCAGTTATGTGCCT |
| CHIKV-R19 | TGTCAGTGAATCCCACCGTC |
| CHIKV-F20 | TGTCCAAAAGGGGAAACTCT |
| CHIKV-R20 | GACAGGAGTGTTGGGTGGTC |
| CHIKV-F21 | TCATCATGCTACTGTATCCTC |
| CHIKV-R21 | AGAACAATCAGGGCTGCCAG |
| CHIKV-F22 | GGCTACAAGCCCTTATTCCG |
| CHIKV-R22 | CGGAGCTTAGCTGATGCAGA |
| CHIKV-F23 | CATACAGGGCTCATACCGCA |
| CHIKV-R23 | TCGCACGACATGTCCGTTAA |
| CHIKV-F24 | GGGTCGTCGACGCGCCCTCTAT |
| CHIKV-R24 | CGTAGCCCTTTGATCTATGGAT |
| CHIKV-F25 | CACACTGCATATAGCAAATACG |
| CHIKV-R25 | ATCTCCTACGTCCCTGTGGG |
