# Supplementary material for: Chikungunya virus in dengue-suspected patients: Molecular evidence from the 2019 outbreak in Yangon, Myanmar
Source: PLoS Negl Trop Dis. 2026 May 4;20(5):e0014258. doi: 10.1371/journal.pntd.0014258 (PMC13138656; doi:10.1371/journal.pntd.0014258)
Supplement: S3 Table — Distribution of categorical demographic and clinical variables across infection groups. Comparisons were exploratory. (DOCX) [file pntd.0014258.s004.docx]

**S3 Table. Descriptive comparison of demographic, clinical and laboratory profiles of dengue, chikungunya, and co-detection cases in dengue-suspected patients in Yangon Myanmar**

| **Variables** | **DENV infection**  **n (%)** | **CHIKV infection**  **n (%)** | **Co-detection**  **n (%)** | **Total**  **n (%)** | **P-value** |
| --- | --- | --- | --- | --- | --- |
| **Age groups** |  |  |  |  | 0.924 |
| ≤5 | 78 (87.6) | 5 (5.6) | 6 (6.7) | 89 (100.0) |  |
| 6-15 | 130 (86.7) | 10 (6.7) | 10 (6.7) | 150 (100.0) |  |
| 16-45 | 10 (90.9) | 0 (0.0) | 1 (9.1) | 11 (100.0) |  |
| **Gender** |  |  |  |  | 0.744 |
| Female | 100 (85.5) | 8 (6.8) | 9 (7.7) | 117 (100.0) |  |
| Male | 118 (88.7) | 7 (5.3) | 8 (6.0) | 133 (100.0) |  |
| **Clinical diagnosis** |  |  |  |  | 0.940 |
| DWoWS | 52 (85.2) | 5 (8.2) | 4 (6.6) | 61 (100.0) |  |
| DWWS | 154 (88.0) | 9 (5.1) | 12 (6.9) | 175 (100.0) |  |
| Severe dengue | 12 (85.7) | 1 (7.1) | 1 (7.1) | 14 (100.0) |  |
| **NS1 test** |  |  |  |  | **<0.001** |
| Negative | 55 (67.1) | 15 (18.3) | 12 (14.6) | 82 (100.0) |  |
| Positive | 163 (97.0) | 0 (0.0) | 5 (3.0) | 168 (100.0) |  |
| **Hess test** |  |  |  |  | **0.001** |
| Yes | 218 (87.6) | 15 (6.0) | 16 (6.4) | 249 (100.0) |  |
| **Headache** |  |  |  |  | 0.117 |
| Yes | 14 (82.4) | 0 (0.0) | 3 (17.6) | 17 (100.0) |  |
| **Muscle pain** |  |  |  |  | 0.108 |
| Yes | 11 (73.3) | 1 (6.7) | 3 (20.0) | 15 (100.0) |  |
| **Joint pain** |  |  |  |  | **0.009** |
| Yes | 6 (60.0) | 1 (10.0) | 3 (30.0) | 10 (100.0) |  |
| **Rash** |  |  |  |  | 0.598 |
| Yes | 6 (85.7) | 0 (0.0) | 1 (14.3) | 7 (100.0) |  |
| **Vomit** |  |  |  |  | 0.152 |
| Yes | 77 (81.9) | 8 (8.5) | 9 (9.6) | 94 (100.0) |  |
| **Diarrhea** |  |  |  |  | 0.637 |
| Yes | 6 (100.0) | 0 (0.0) | 0 (0.0) | 6 (100.0) |  |
| **Abdominal pain** |  |  |  |  | 0.197 |
| Yes | 79 (90.8) | 2 (2.3) | 6 (6.9) | 87 (100.0) |  |
| **Bleeding** |  |  |  |  | 0.800 |
| Yes | 3 (100.0) | 0 (0.0) | 0 (0.0) | 3 (100.0) |  |
| **Malena** |  |  |  |  | **0.002** |
| Yes | 7 (100.0) | 0 (0.0) | 0 (0.0) | 7 (100.0) |  |
| **Hepatomegaly** |  |  |  |  | **0.042** |
| Yes | 43 (84.3) | 1 (2.0) | 7 (13.7) | 51 (100.0) |  |
| **CHIKV diagnosis** |  |  |  |  | **<0.001** |
| Negative | 218 (100.0) | 0 (0.0) | 0 (0.0) | 218 (100.0) |  |
| Positive | 0 (0.0) | 15 (46.9) | 17 (53.1) | 32 (100.0) |  |
| **Type of DENV Infection** |  |  |  |  | **<0.001** |
| Past flavi-infection | 2 (15.4) | 11 (84.6) | 0 (0.0) | 13 (100.0) |  |
| Primary | 60 (90.9) | 4 (6.1) | 2 (3.0) | 66 (100.0) |  |
| Secondary | 156 (91.2) | 0 (0.0) | 15 (8.8) | 171 (100.0) |  |
