# Supplementary material for: Chikungunya virus in dengue-suspected patients: Molecular evidence from the 2019 outbreak in Yangon, Myanmar
Source: PLoS Negl Trop Dis. 2026 May 4;20(5):e0014258. doi: 10.1371/journal.pntd.0014258 (PMC13138656; doi:10.1371/journal.pntd.0014258)
Supplement: S4 Table — (A) Nonstructural proteins and (B) Structural protein. (A) Shows the non-structural proteins amino acid’s differences; (B) Shows the structural proteins amino acid’s differences. The dashes indicate the conserved residues. (DOCX) [file pntd.0014258.s005.docx]

**S4 Table. Alignment of amino acid sequences of CHIKV isolates in this study in comparison with Myanmar reference strains.**

**(A) Nonstructural proteins**

|  | Protein name | NSP1 | | | | | NSP2 | | | | | |
| --- | --- | --- | --- | --- | --- | --- | --- | --- | --- | --- | --- | --- |
|  | Protein position | 17 | 290 | 314 | 496 | 517 | 665 | 680 | 1027 | 1030 | 1132 | 1328 |
|  | Specific aa position | 17 | 290 | 314 | 496 | 517 | 130 | 145 | 492 | 495 | 597 | 793 |
| Reference Strain | KF590567 | V | I | M | E | Q | H | E | I | N | P | V |
| CHIKV isolates in this study | PV664498 | A | - | - | - | - | Y | D | - | S | - | A |
|  | PV683444 | A | - | - | - | - | Y | D | - | S | - | A |
|  | PV683445 | A | - | - | V | - | Y | D | - | S | - | A |
|  | PV683446 | A | - | - | - | - | Y | D | - | S | H | A |
|  | PV683447 | A | - | - | - | - | Y | D | - | S | - | A |
|  | PV683448 | A | - | - | V | R | Y | D | - | S | - | A |
|  | PV683449 | A | - | - | - | - | Y | D | - | S | - | A |
|  | PV683450 | A | - | - | - | - | Y | D | - | S | - | A |
|  | PV683451 | A | - | - | - | - | Y | D | - | S | - | A |
|  | PV683452 | A | - | - | - | - | Y | D | - | S | - | A |
|  | PV683453 | A | V | L | - | - | Y | D | - | S | - | A |
|  | PV683454 | A | V | - | - | - | Y | D | - | S | - | A |
|  | PV683455 | A | V | - | - | - | Y | D | M | S | - | A |
|  | PV683456 | A | - | - | - | - | Y | D | - | S | - | A |
|  | PV683457 | A | - | - | - | - | Y | D | - | S | - | A |

The (–) indicates conserved residues.

|  | Protein name | NSP3 | | | | NSP4 | | | | |
| --- | --- | --- | --- | --- | --- | --- | --- | --- | --- | --- |
|  | Protein position | 1550 | 1673 | 1690 | 1705 | 1899 | 1918 | 1945 | 1948 | 2350 |
|  | Specific aa position | 217 | 340 | 357 | 372 | 36 | 55 | 82 | 85 | 487 |
| Reference Strain | KF590567 | H | L | P | D | Y | S | S | R | M |
| CHIKV isolates in this study | PV664498 | - | - | - | E | - | N | R | G | - |
|  | PV683444 | - | - | - | E | H | N | R | G | V |
|  | PV683445 | - | - | - | E | - | N | R | G | V |
|  | PV683446 | - | - | - | E | - | N | R | G | V |
|  | PV683447 | - | - | - | E | - | N | R | G | V |
|  | PV683448 | - | - | - | E | - | N | R | G | V |
|  | PV683449 | - | - | - | E | - | N | R | G | V |
|  | PV683450 | - | - | - | E | - | N | R | G | - |
|  | PV683451 | - | P | - | E | - | N | R | G | V |
|  | PV683452 | - | - | - | E | - | N | R | G | V |
|  | PV683453 | Y | - | - | E | - | N | R | G | - |
|  | PV683454 | Y | - | - | E | - | N | R | G | - |
|  | PV683455 | Y | - | T | E | - | N | R | G | - |
|  | PV683456 | - | - | - | E | - | N | R | G | V |
|  | PV683457 | - | - | - | E | - | N | R | G | V |

**S4 Table. Alignment of amino acid sequences of CHIKV isolates in this study in comparison with Myanmar reference strains.**

**(B) Structural proteins**

|  | Protein name | C | E3 | E2 | | | | | | E1 | | | | |
| --- | --- | --- | --- | --- | --- | --- | --- | --- | --- | --- | --- | --- | --- | --- |
|  | Protein position | 73 | 264 | 432 | 530 | 577 | 589 | 637 | 702 | 964 | 1020 | 1035 | 1126 | 1160 |
|  | Specific position | 73 | 3 | 107 | 205 | 252 | 264 | 312 | 377 | 155 | 211 | 226 | 317 | 351 |
| Reference Strain | KF590567 | K | V | K | G | Q | V | M | I | T | K | V | I | Q |
| CHIKV isolates in this study | PV664498 | R | A | - | S | K | A | - | - | - | E | A | V | - |
|  | PV683444 | R | A | - | S | K | A | - | - | - | E | A | V | - |
|  | PV683445 | R | A | - | S | K | A | - | - | - | E | A | V | - |
|  | PV683446 | R | A | - | S | K | A | - | - | - | E | A | V | - |
|  | PV683447 | R | A | - | S | K | A | - | - | - | E | A | V | - |
|  | PV683448 | R | A | - | S | K | A | - | - | - | E | A | V | - |
|  | PV683449 | R | A | - | S | K | A | - | - | - | E | A | V | - |
|  | PV683450 | R | A | - | S | K | A | - | T | - | E | A | V | - |
|  | PV683451 | R | A | Q | S | K | A | - | - | - | E | A | V | - |
|  | PV683452 | R | A | - | S | K | A | - | - | - | E | A | V | R |
|  | PV683453 | R | A | - | S | K | A | - | - | - | E | A | V | - |
|  | PV683454 | R | A | - | S | K | A | - | - | - | E | A | V | - |
|  | PV683455 | R | A | - | S | K | A | - | - | I | E | A | V | - |
|  | PV683456 | R | A | - | S | K | A | I | - | - | E | A | V | - |
|  | PV683457 | R | A | - | S | K | A | - | - | - | E | A | V | - |
