# Supplementary material for: Chikungunya virus in dengue-suspected patients: Molecular evidence from the 2019 outbreak in Yangon, Myanmar
Source: PLoS Negl Trop Dis. 2026 May 4;20(5):e0014258. doi: 10.1371/journal.pntd.0014258 (PMC13138656; doi:10.1371/journal.pntd.0014258)
Supplement: S5 Table — (B) Mutation Analysis of CHIKV isolates positives from dengue-suspected patients in Yangon, Myanmar 2019. A comprehensive list of amino-acid substitutions identified in the 15 CHIKV isolates, including functional insights, biological impacts, and supporting literature references. (DOCX) [file pntd.0014258.s006.docx]

**S5 Table. (A) Distribution of CHIKV Mutations Across Non-Structural and Structural Proteins from Whole Genome Sequences of Isolates in Dengue-Suspected Patients, Yangon, Myanmar (2019)**

| **CHIKV NONSTRUCTURAL PROTEIN n=4 (n=20 mutations)** | | |  |
| --- | --- | --- | --- |
| **Protein Name** | **Protein Key role** | **Mutation found in our study isolates** |  |
| NSP1 (n=5) | Crucial role in viral RNA capping, which is important for efficient translation and replication of the viral genome | NSP1-V17A NSP1-I290V NSP1-M314L NSP1-E496V NSP1-Q517R |  |
|  |  |  |  |
|  |  |  |  |
|  |  |  |  |
|  |  |  |  |
| NSP2 (n=6) | Crucial role in viral RNA replication, inhibiting the host's antiviral responses | NSP2-H130Y NSP2-E145D NSP2-I492M  NSP2-N495S NSP2-P597H NSP2-V793A |  |
|  |  |  |  |
|  |  |  |  |
|  |  |  |  |
|  |  |  |  |
|  |  |  |  |
| NSP3 (n=4) | Critical role in viral replication, RNA synthesis, and interactions with both viral and cellular components | NSP3-H217Y NSP3-L340P NSP3-P357T NSP3-D372E |  |
|  |  |  |  |
|  |  |  |  |
|  |  |  |  |
| NSP4 (n=5) | Act as RNA-dependent RNA polymerase essential for genome replication | NSP4-Y36H NSP4-S55N NSP4-S82R NSP4-R85G NSP4-M487V |  |
|  |  |  |  |
|  |  |  |  |
|  |  |  |  |
|  |  |  |  |
| **CHIKV STRUCTURAL PROTEIN n=5 (n=13 mutations)** | | |  |
| **Protein Name** | **Protein Key role** | **Mutation found in our study isolates** |  |
| Capsid (n=1) | Packages the viral RNA genome and mediates nucleocapsid assembly | CAPSID-K73R |  |
| E3 (n=1) | Functions as a chaperone during viral glycoprotein processing and protects E2 from premature fusion | E3-V3A |  |
| E2 (n=6) | Mediates receptor binding and entry into host cells | E2-K107Q E2-G205S E2-Q252K E2-V264A  E2-M312I E2-I377T |  |
|  |  |  |  |
|  |  |  |  |
|  |  |  |  |
|  |  |  |  |
|  |  |  |  |
| 6K | Virus assembly and budding | **─** |  |
| E1 (n=5) | Responsible for membrane fusion during viral entry and required for infectivity | E1-T155I  E1-K211E E1-V226A E1-I317V E1-Q351R |  |
|  |  |  |  |
|  |  |  |  |
|  |  |  |  |
|  |  |  |  |

**S5 Table. (B) Mutation Analysis of CHIKV isolates positives from dengue-suspected patients in Yangon, Myanmar 2019.**

| **N** | **Virus isolate ID in our study** | **Mutation** | **Functional Insight** | **Biological Impact** | **Known* or Unknown**** | **Reference** |
| --- | --- | --- | --- | --- | --- | --- |
| 1 | ALL 15 isolates | NSP1-V17A | Function currently unknown. | Biological effect remains uncharacterized. | Unknown | - |
| 2 | 3 isolates: PV683453, PV683454, PV683455 | NSP1-I290V | Located in the hydrophobic core of the viral RNA capping enzyme, nsP1 | may have slight effects on protein stability but is not expected to significantly impair its function due to its conservative nature and buried position. | Known | [7,48] |
| 3 | PV683453 | NSP1-M314L | Function currently unknown. | Biological effect remains uncharacterized. | Unknown | - |
| 4 | 2 isolates: PV683445, PV683448 | NSP1-E496V | Function currently unknown. | Biological effect remains uncharacterized. | Unknown | - |
| 5 | PV683448 | NSP1-Q517R | Function currently unknown. | Biological effect remains uncharacterized. | Unknown | - |
| 6 | ALL 15 isolates | NSP2-H130Y | Suggested role in viral replication (helicase domain) | May reflect viral adaptation during outbreak | Known | [30,43,53] |
| 7 | ALL 15 isolates | NSP2-E145D | May influence RNA unwinding during replication. | Clade marker, possible adaptation signature. Potential role in viral replication remains speculative. | Known | [30,43,53] |
| 8 | PV683455 | NSP2-I492M | potential role in polyprotein processing. | Effect on viral replication. Pathogenesis remains unknown | Known | [42] |
| 9 | ALL 15 isolates | NSP2-N495S | Maintains hydrophilic character. | Subclade-defining mutation. | Known | [30,43,53] |
| 10 | PV683446 | NSP2-P597H | Function currently unknown. | Biological effect remains uncharacterized. | Unknown | - |
| 11 | ALL 15 isolates | NSP2-V793A | May influence nsP2 function. | Potentially affects viral replication but impact remains uncharacterized | known | [45] |
| 12 | 3 isolates: PV683453, PV683454, PV683455 | NSP3-H217Y | Function currently unknown. | Biological effect remains uncharacterized. | known | [5] |
| 13 | PV683451 | NSP3-L340P | Function currently unknown. | Biological effect remains uncharacterized. | Unknown | - |
| 14 | PV683455 | NSP3-P357T | Function currently unknown. | Biological effect remains uncharacterized. | Unknown | - |
| 15 | ALL 15 isolates | NSP3-D372E | May affect immune evasion or protein folding | Could alter antibody binding and recognition; linked to outbreak adaptation | Known | [45] |
| 16 | PV683444 | NSP4-Y36H | Function currently unknown. | Biological effect remains uncharacterized. | Unknown | - |
| 17 | 10 isolates: PV683444, PV683445, PV683446, PV683447, PV683448, PV683449, PV683451, PV683452, PV683456, PV683457 | NSP4-S55N | Impacts replication fidelity by affecting polymerase activity. | May alter viral replication efficiency. | Known | [43] |
| 18 | ALL 15 isolates | NSP4-S82R | Reversion | Biological effect remains uncharacterized. | Known | [44] |
| 19 | ALL 15 isolates | NSP4-R85G | lineage-defining mutation within the IOL of the ECSA genotype. | Biological effect remains uncharacterized. | Known | [43] |
| 20 | ALL 15 isolates | NSP4-M487V | Function currently unknown. | Biological effect remains uncharacterized. | Known | [30,43,53] |
| 21 | ALL 15 isolates | CAPSID-K73R | Subclade marker | Suggested as part of a set of mutations that may enhance viral fitness, transmission, or pathogenesis | Known | [30,53] |
| 22 | ALL 15 isolates | E3-V3A | Function currently unknown. | functional effects remain uncharacterized | known | [5] |
| 23 | PV683451 | E2-K107Q | Function currently unknown. | Biological effect remains uncharacterized. | Unknown | - |
| 24 | ALL 15 isolates | E2-G205S | Alter antibody binding and recognition due to their position | Potentially contributes to immune evasion, Epidemiological marker | Known | [30,53] |
| 25 | ALL 15 isolates | E2-Q252K | Associated *with Ae. albopictus* adaptation. | indicates a shift towards *Ae. Aegypti* adaptation | Known | [30,53] |
| 26 | ALL 15 isolates | E2-V264A | Contributes to enhanced replication. Higher fitness for *Ae. aegypti* | Improves infectivity, dissemination, and transmission in *Ae. aegypti* | Known | [47] |
| 27 | PV683456 | E2-M312I | May influence immune recognition | Biological effect remains uncharacterized. | Unknown | - |
| 28 | PV683450 | E2-I377T | Potential role in viral evolution | Biological effect remains uncharacterized. | Known | [49] |
| 29 | PV683455 | E1-T155I | may affect viral fusion or stability | Biological effect remains uncharacterized. | Known | [50] |
| 30 | ALL 15 isolates | E1-K211E | Enhances vector competence. | Increases transmissibility in *Ae. aegypti* and viral replication. | Known | [47] |
| 31 | ALL 15 isolates | E1-V226A | Revert from A226V variant. Higher fitness for *Ae. aegypti* | Enhances adaptation to *Ae. aegypti***.** | Known | [54] |
| 32 | ALL 15 isolates | E1-I317V | may support viral fitness. | May enhance infectivity or stability. | Known | [30,53] |
| 33 | PV683452 | E1-Q351R | Function currently unknown. | Biological effect remains uncharacterized. | Unknown | - |

*Previously published, **Not detected/published in previous research (New from our study).
