# Supplementary material for: Chikungunya virus in dengue-suspected patients: Molecular evidence from the 2019 outbreak in Yangon, Myanmar
Source: PLoS Negl Trop Dis. 2026 May 4;20(5):e0014258. doi: 10.1371/journal.pntd.0014258 (PMC13138656; doi:10.1371/journal.pntd.0014258)
Supplement: S6 Table — The Fisher’s exact test results for associations between amino-acid mutations and disease severity (DWoWS vs DWWS). The adjusted p-values were computed using the Benjamini-Hochberg method. (DOCX) [file pntd.0014258.s007.docx]

**S6 Table. Association between CHIKV amino-acid mutations and disease severity**

| **Mutation** | **DWoWS-No mutation** | **DWWS-No mutation** | **DWoWS-with mutation** | **DWWS-with mutation** | **Total (n)** | **Odds ratio** | **p-value** | **Adj p (BH)** |
| --- | --- | --- | --- | --- | --- | --- | --- | --- |
| E1-L351R | 3 | 11 | 1 | 0 | 15 |  | 0.27 | 1 |
| NSP1-Q517R | 3 | 11 | 1 | 0 | 15 |  | 0.27 | 1 |
| NSP4-Y36H | 3 | 11 | 1 | 0 | 15 |  | 0.27 | 1 |
| NSP1-E496V | 3 | 10 | 1 | 1 | 15 | 3.33 | 0.48 | 1 |
| NSP1-I290V | 4 | 8 | 0 | 3 | 15 | 0 | 0.52 | 1 |
| NSP3-H217Y | 4 | 8 | 0 | 3 | 15 | 0 | 0.52 | 1 |
| E1-T155I | 4 | 10 | 0 | 1 | 15 | 0 | 1 | 1 |
| E2-I377T | 4 | 10 | 0 | 1 | 15 | 0 | 1 | 1 |
| E2-K107Q | 4 | 10 | 0 | 1 | 15 | 0 | 1 | 1 |
| E2-M312I | 4 | 10 | 0 | 1 | 15 | 0 | 1 | 1 |
| NSP1-M314L | 4 | 10 | 0 | 1 | 15 | 0 | 1 | 1 |
| NSP2-I492M | 4 | 10 | 0 | 1 | 15 | 0 | 1 | 1 |
| NSP2-P597H | 4 | 10 | 0 | 1 | 15 | 0 | 1 | 1 |
| NSP3-L340P | 4 | 10 | 0 | 1 | 15 | 0 | 1 | 1 |
| NSP3-P357T | 4 | 10 | 0 | 1 | 15 | 0 | 1 | 1 |
| NSP4-S55N | 1 | 4 | 3 | 7 | 15 | 1.71 | 1 | 1 |

DWoWS: Dengue without warning signs, DWWS: Dengue with warning signs. Fisher’s exact test used to evaluate associations between amino-acid mutations and disease severity. Each mutation row shows the distribution of cases with and without the mutation in both severity categories from our isolates, for each mutation, a 2×2 contingency table was constructed comparing the presence/absence of the mutation with disease severity. Odds ratio calculated by Fisher’s exact test. Adjusted p-values correspond to the Benjamini-Hochberg method.
