# Supplementary figures and images for: Chikungunya virus in dengue-suspected patients: Molecular evidence from the 2019 outbreak in Yangon, Myanmar
Source: PLoS Negl Trop Dis. 2026 May 4;20(5):e0014258. doi: 10.1371/journal.pntd.0014258 (PMC13138656; doi:10.1371/journal.pntd.0014258)

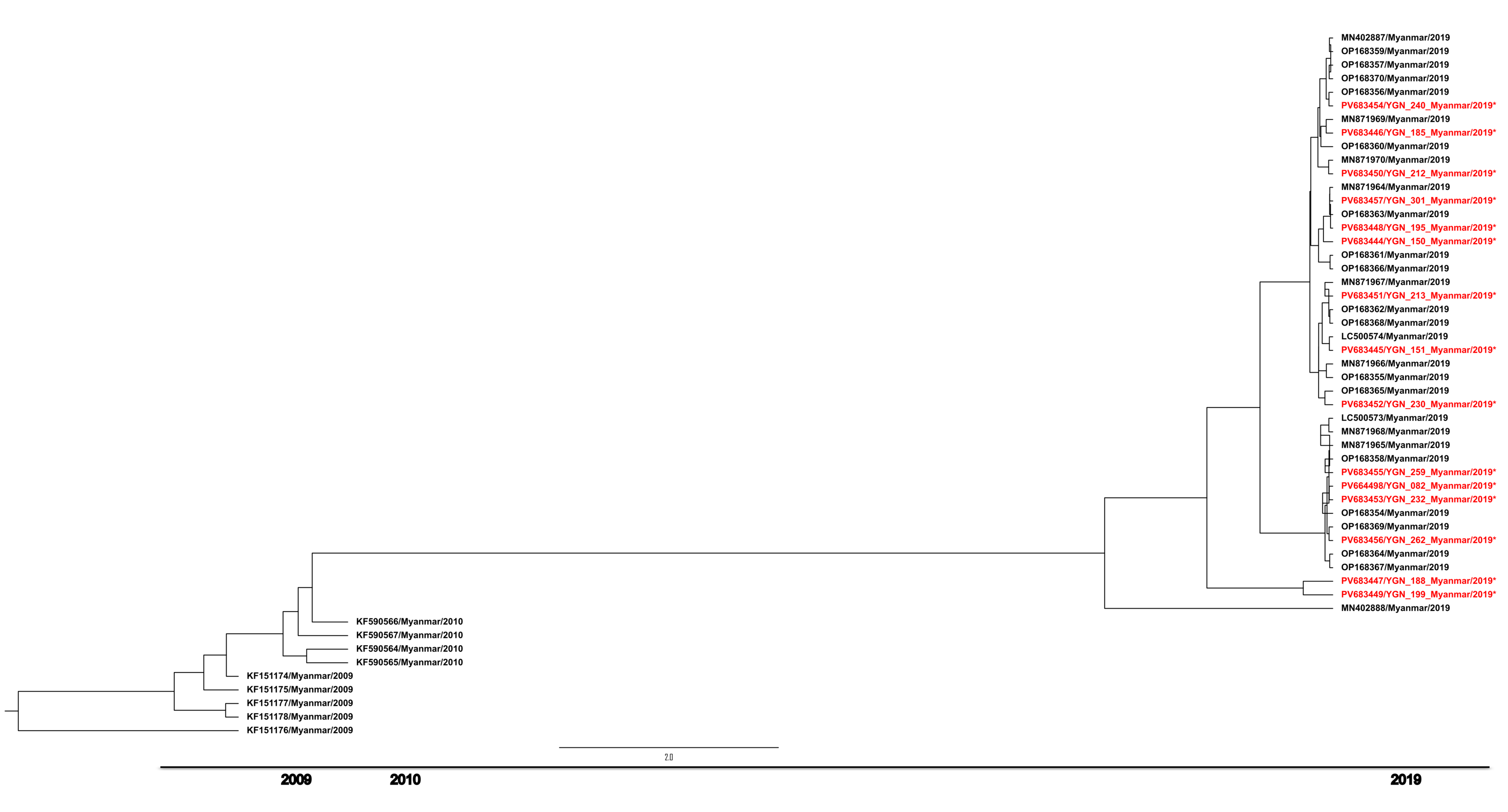

Supplement: S1 Fig — A time-scaled Bayesian phylogenetic tree generated in BEAST v1.10.4 illustrating the temporal clustering of 2009, 2010, and 2019 Myanmar isolates within the ECSA lineage (red: study isolates). (TIF) [file pntd.0014258.s008.tif]

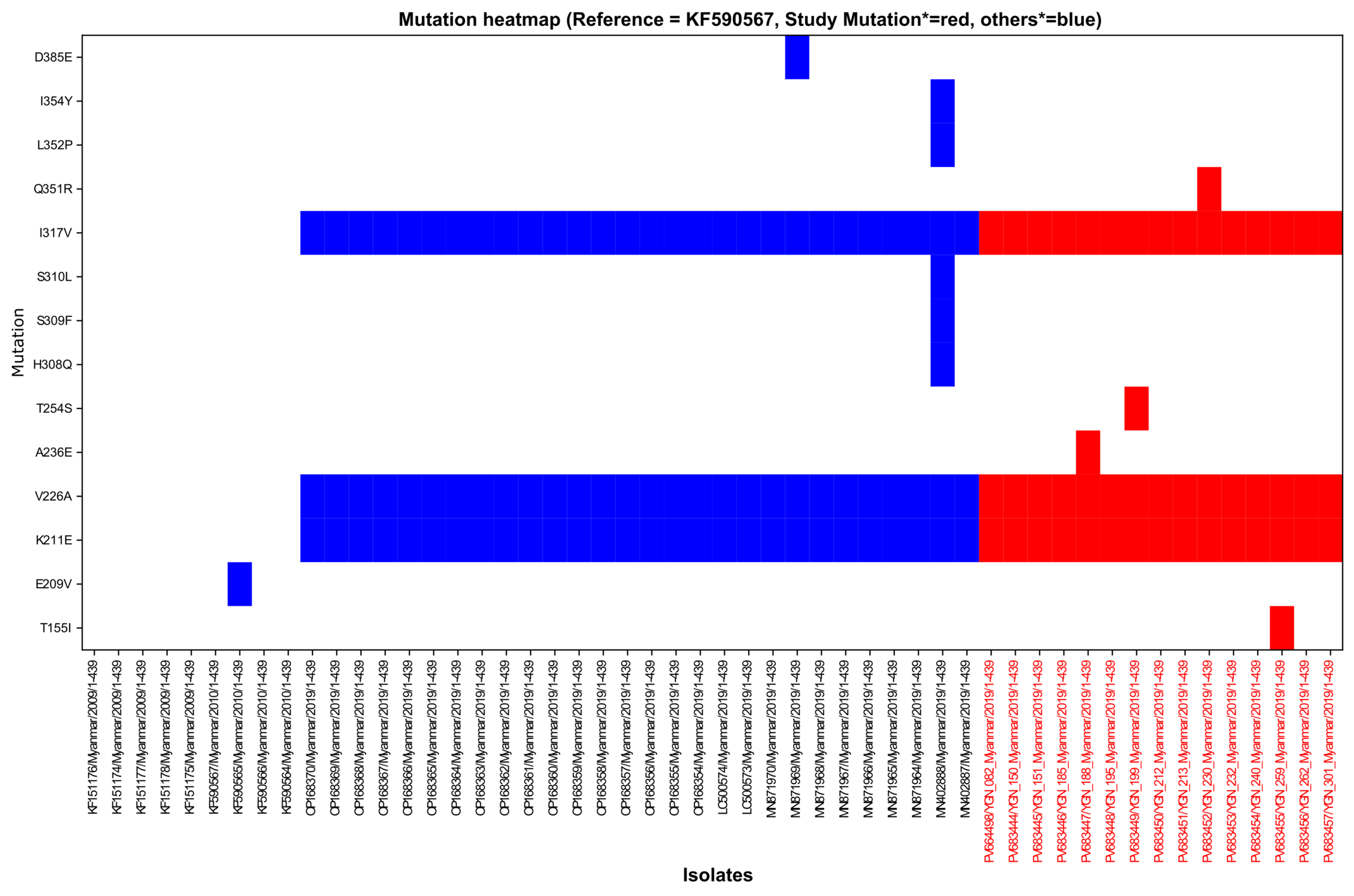

Supplement: S2 Fig — The heatmap summarizing amino-acid substitutions across Myanmar isolates highlighting conserved and variable sites within the ECSA genotype (red: study 15 isolates). (TIF) [file pntd.0014258.s009.tif]

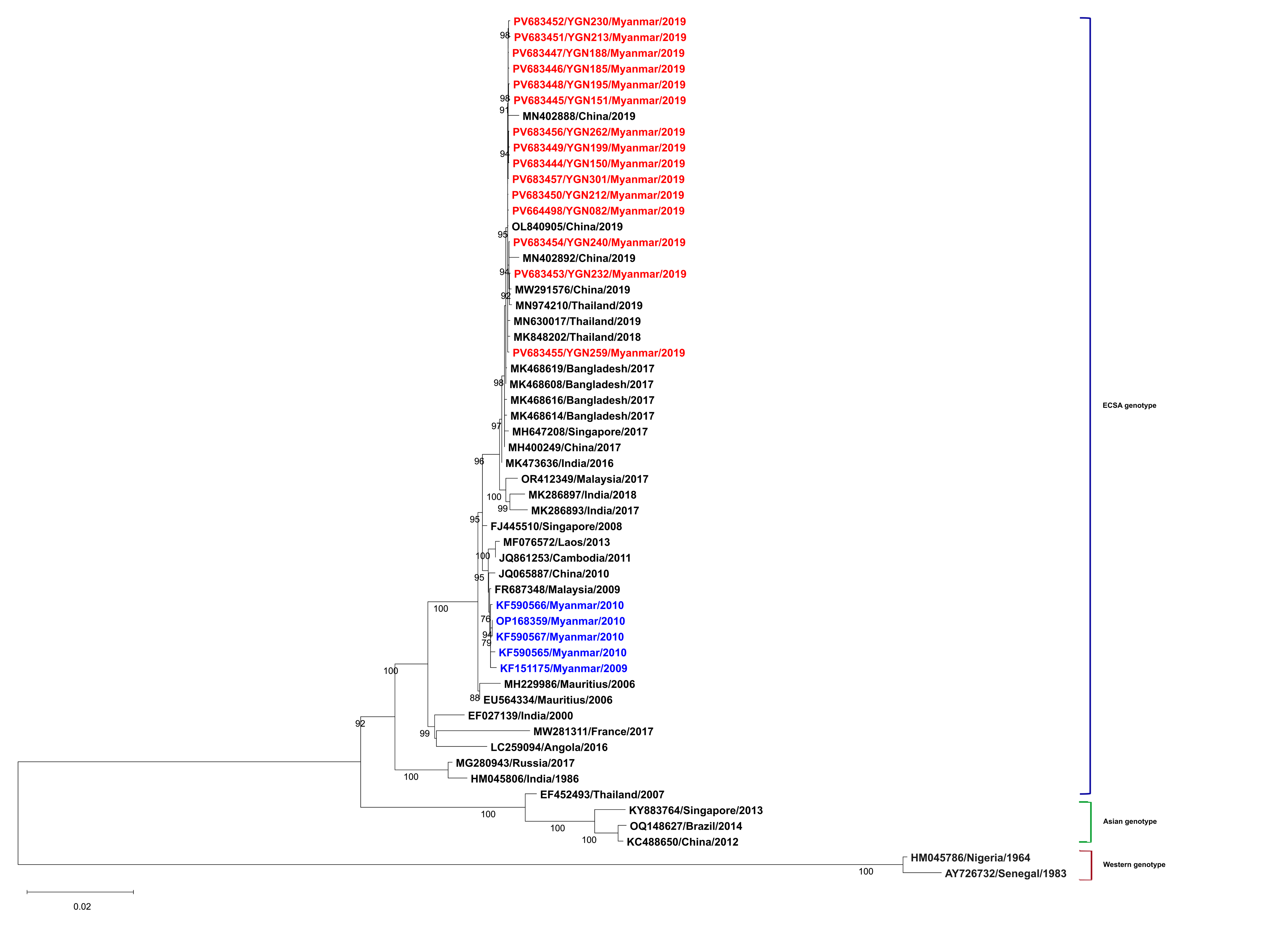

Supplement: S3 Fig — The Maximum-likelihood tree (IQ-TREE 2) constructed from 461 E1 gene sequences, including 15 Myanmar isolates from this study and 446 global strains. Myanmar isolates are shown in red (study isolates) and blue (previous Myanmar strains). (TIF) [file pntd.0014258.s010.tif]
